# The MicroRNA-106a/20b Strongly Enhances the Antitumour Immune Responses of Dendritic Cells Pulsed with Glioma Stem Cells by Targeting STAT3

**DOI:** 10.1155/2022/9721028

**Published:** 2022-09-15

**Authors:** Hui Zhou, Chengmei Sun, Cong Li, Shiting Hua, Feng Li, Ruichun Li, Dongpeng Cai, Yuxi Zou, Yingqian Cai, Xiaodan Jiang

**Affiliations:** ^1^Neurosurgery Center, The National Key Clinical Specialty, The Engineering Technology Research Center of Education Ministry of China on Diagnosis and Treatment of Cerebrovascular Disease, Guangdong Provincial Key Laboratory on Brain Function Repair and Regeneration, The Neurosurgery Institute of Guangdong Province, Zhujiang Hospital, Southern Medical University, Guangzhou 510282, China; ^2^Key Laboratory of Mental Health of the Ministry of Education, Guangdong-Hong Kong-Macao Greater Bay Area Center for Brain Science and Brain-Inspired Intelligence, Southern Medical University, Guangzhou 510515, China; ^3^Department of Neurosurgery, The First Affiliated Hospital of Guangdong Pharmaceutical University, Guangzhou 510080, China

## Abstract

**Background:**

Evaluate the effect of the miRNA-106a/20b on the efficacy of DCs pulsed with GSCs in activating GSC-specific T cell responses.

**Methods:**

We cultured GSCs and prepared GSC antigen lysates by apoptosis. Then, immature DCs were pulsed with GSC antigen lysates in vitro. STAT3 levels in DCs were assessed by Western blotting, and the expression of CD80, CD86, and MHC-II was tested by fluorescence-activated cell sorting. The production and secretion of the cytokines IL-6, IL-12, TNF-*α*, and IL-10 in DCs induced by GSCs were determined by enzyme-linked immunosorbent assay. Finally, the cytotoxic functions of T cells stimulated by GSC-DC fusion cells transfected with a miR-106a/20b mimic in vitro and the antitumour activity in vivo were detected.

**Results:**

We found that the levels of miR-106a/20b were downregulated, but the expression of STAT3 was significantly upregulated. Simultaneously, the inhibition of STAT3 in the fusion cells by STAT3-specific siRNA caused significant upregulation of the expression of CD80, CD86, and MHC-II, and the secretion of the cytokines IL-6 and IL-12 was substantially increased, IL-10 was markedly decreased. These findings revealed that STAT3 is an important regulator of DC maturation. Furthermore, the interactional binding sites between the 3′-untranslated region (3′-UTR) of STAT3 mRNA and miR-106a/20b were predicted by bioinformatics and verified by a dual-luciferase assay. Moreover, the reduction in STAT3 levels in GSC-DCs enhanced the generation of CD8+ T cells and reduced the generation of Foxp3+ regulatory T cells. Meanwhile, the secretion of the T cell cytokine IFN-*γ* was significantly increased. Further research showed that DCs after miR-106a/20b-mimics transfection could promote the inhibition of GSC proliferation by T cells in vitro and suppress tumour growth in vivo.

**Conclusions:**

This study indicted that the miR-106a/20b activation could be one of the important molecular mechanisms leading to enhance antitumour immune responses of GSC-mediated DCs, which downregulated the expression of STAT3 to alleviate its the inhibitory effect.

## 1. Background

Glioma is the most common primary central nervous tumour in humans and has a poor prognosis and high recurrence rate. Despite the development of multidisciplinary treatments, including surgery, chemotherapy, and radiotherapy [1], the median survival time of high-grade glioma (HGG) patients is only approximately 14.6 months, and the five-year survival rates are less than 5% [2]. At present, cellular immunotherapy is considered a novel proven treatment with potential applications in glioma therapy [3].

Dendritic cells (DCs) are central to the immune system. They are the most powerful antigen-presenting cells (APCs) and recognize, capture, process, and present antigens through their surface molecules to activate priming of the T cell response to an “enemy” [4]. Thus, DC-based immunotherapy is a very promising strategy to fight glioma. During the last decade, numerous studies have aimed to develop DCs loaded with tumour-derived peptides ex vivo, which can process and present a broad array of tumour antigens, including tumour-associated antigens, autologous T cells and effectively induce specific T cell immunity [5].

However, cancer stem cells (CSCs), including glioma stem cells (GSCs), are the root of tumour onset, progression, metastasis, and recurrence, as well as drug resistance, and are one of the greatest obstacles to eradicating tumours [6]. Li et al. reported that DC vaccination using lung CSC antigens could effectively induce major histocompatibility complex (MHC) expression, cytokine production, lymphocyte infiltration, and long-term protection against prostate cancer [7]. Therefore, CSCs are expected to be a new more effective therapeutic target for DCs in glioma immunotherapy, which has rarely been reported in the literature. Here, we will focus on the high expression levels of MHC-II, CD80, and CD86 and functionally maturation of DCs targeting GSCs that can activate effector cytotoxic T-lymphocytes that possess antiglioma activity.

In the past, it has been demonstrated that signal transducer and activator of transcription-3 (STAT3) is a key transcription factor involved in immunoregulation [8]. High levels of STAT3 can augment the release of tolerogenic mediators, allowing tumours to escape immune detection and decreasing immunostimulatory molecule transcription, thereby reducing innate and adaptive antitumour immunity [9]. Its activation has been shown to suppress DC maturation, and STAT3-deficient dendritic cells displayed heightened immunological activity, including increased cytokine production, resistance to IL-10-mediated inhibition and Ag-dependent T cell activation [10]. However, the mechanism by which upstream signals regulate the transcriptional expression of STAT3 in DCs recognizing tumour antigens, especially GSC antigens, remains poorly understood.

It is well known that microRNAs (miRNAs), as a class of regulators, are widely expressed in animal cells and play a critical functional role in glioma cellular differentiation, proliferation, metabolism, apoptosis, signal transduction, and immune function, such as in monocytes and DCs [11]. It has been reported that the miR-4458 overexpression enhanced antitumour immunity by targeting STAT3 to block the PD-L1/PD-1 pathway [12]. Exploring miRNAs with the ability to inhibit STAT3 gene expression would help in developing a reasonable glioma immunotherapy. Recent studies have implicated that miR-106a deficiency promoted Treg induction, increased IL-10 secretion, and suppressed the inflammatory response in vitro [13]. Notably, miR-106a is also a proven biomarker for cancer and an independent prognostic marker in patients with glioblastoma [14]. Interestingly, miR-20b, which is near miR-106a, within a 1kb distance, has been reported to be a downregulated miRNA that suppresses Th17 differentiation and reduces the severity of the autoimmune inflammatory response by targeting ROR*γ*t and STAT3 [15]. Taken together, the results suggest that the relationship between miR-106a/20b and STAT3 signalling in the maturation of DCs recognizing GSC antigens deserves further study.

In the present study, we utilized bone marrow-derived DCs (BMDCs), pulsed with total apoptotic GSC mRNA isolated from GL261 cells, to analyze the expression of miR-106a/20b and its effect on STAT3 in inducing DC maturation and T cell differentiation. These findings may provide a novel strategy for glioma immunotherapy targeting GSCs.

## 2. Methods

### 2.1. Experimental Mice and Cell Culture

Mice of the C57 BL/6 strain that were between six and eight weeks old and weighed between 22 and 26 grams each were obtained from the Institute of Laboratory Animals Science (No.1103221911009975). The animals were bred under specific pathogen-free conditions, with food and water freely supplied by The First Affiliated Hospital of Guangdong Pharmaceutical University. The protocols used in the research were approved by the Animal Care and Use Committee of The First Affiliated Hospital of Guangdong Pharmaceutical University (NO.2017076). Every effort was made to reduce the amount of animals that were put through unnecessary pain and the total number of animals employed.

A murine glioma cell line (GL261) was purchased from iCell Bioscience Inc. (Shanghai, China, No.202009034298601). GL261 cells were cultured in DMEM containing 10% FBS (Gibco, USA), 2 mM glutamine, and 0.1% penicillin (100 U/mL) and streptomycin (100 *μ*g/mL) at 37°C with 5% CO_2_.

Immature DCs (imDCs) were isolated from the bone marrow of C57BL/6 mice as previously described [16] and seeded on non-tissue-culture-treated 100 mm dishes in RPMI-1640 (Gibco, USA) medium at 37°C with 5% CO_2_. Floating cells were removed on day 3. On the 5th day of culture, some nonadherent imDCs were collected by gentle pipetting for phenotypic analysis. On the 7th day of culture, imDCs were collected for GSC antigen loading procedures.

T-lymphocytes were obtained from C57BL/6 splenocytes as previously described [17]. Magnetic-activated cell sorting (MACS) of CD3-positive T-lymphocytes was performed using the EasySep™ Mouse T Cell Enrichment Kit. Then, the purity of the T-lymphocytes was determined by flow cytometry using FITC-labelled antiCD3 (1 : 100, BD Biosciences, USA). The isolated T-lymphocytes were resuspended in RPMI-1640 medium and cultured in culture dishes at 37°C with 5% CO_2_.

### 2.2. GSC Acquisition and Antigen Preparation

GSCs were obtained according to previous studies [18] by growing GL261 in serum-free DMEM/F12 medium (HyClone, USA) supplemented with 20 ng/mL murine bFGF (PeproTech, USA) and 20 ng/mL EGF (PeproTech, USA) at 37°C with 5% CO_2_. The culture medium was refreshed by 50% every 2-3 days. The cells were passaged when they reached 90% confluence. Spherical cells at the third passage (P3) were examined for the expression of CD133 by immunofluorescence staining. After three days, the expression of GFAP in the spheres was detected by an immunofluorescence assay.

We extracted GSC antigens from apoptotic lysates. GSCs of the third passage (P3) were digested with 0.05% trypsin (HyClone, USA) for 5 min and processed overnight with hydrogen peroxide (100 *μ*M),which is known to induce apoptosis. In order to evaluate apoptosis, an apoptosis assay kit manufactured by Beyotime in China was utilized. Then, GSCs were stained with FITC-Annexin V/PI (KeyGen Biotech, China) for 15 min, and apoptotic antigen labelled with FITC-Annexin V was enriched by fluorescence-activated cell sorting (FACS; BD FACSAria III, USA). The purity of these apoptotic GSCs was verified by flow cytometry test. After sorting, the enriched apoptotic GSCs were added to imDCs at a 3 : 1 (stimulator : responder) ratio in RPMI-1640 medium and incubated for 24 h at 37°C with 5% CO_2_.

### 2.3. Immunofluorescence Staining

After 20 minutes of incubation at room temperature with paraformaldehyde at a concentration of 4%, the cells were permeabilized with 0.05 percent Triton X-100 dissolved in Phosphate Buffered Saline (PBS). The cells were treated at 4 degrees Celsius overnight with antiCD133 (1 : 200; eBioscience, USA) and antiGFAP (1 : 200; eBioscience, USA) antibodies after being blocked for one hour with bovine serum albumin dissolved in PBS at a concentration of five percent bovine serum albumin for one hour. After removing any excess antibody, the cells were washed with PBS and then subjected to an incubation with a secondary antibody that was conjugated to PE-labelled goat antimouse IgG (1 : 200; Invitrogen, USA). Then, a fluorescence microscope was used to view the cells after they had been counterstained with DAPI (made by Beyotime in China).

### 2.4. Flow Cytometry Analysis

As previously described [19], each group's DCs and T cells that were going to be examined in the coculture system were first collected, then washed twice with PBS after being digested with 0.25 percent trypsin. After this, the cells were tested. Following centrifugation, the supernatant was discarded, and the cells were resuspended in Eppendorf (EP) tubes at a concentration of 2 105 cells per 100 *μ*L. Then, l *μ*L of primary antibodies was added to the EP tube, including antimouse CD133 antibody (BioLegend, USA), antimouse CD11c antibody (BioLegend, USA), antimouse CD80 antibody (BioLegend, USA), antimouse CD86 antibody (BioLegend, USA), antimouse MHC-II antibody (BioLegend, USA), antimouse CD4 antibody (BioLegend, USA), antimouse CD8 antibody (BioLegend, USA), and antimouse FOXp antibody (BioLegend, USA). Following a thirty minute incubation period in the dark at a temperature of four degrees Celsius, the cells were resuspended in 0.2 milliliters of PBS before being washed twice with PBS. The phenotypic changes in DCs and T cells were identified with the help of flow cytometry (a BD FACSCalibur), and the analysis of the experiment's results was performed with the FlowJo software tool (FlowJo™ v10.7, http://www.flowjo.com).

### 2.5. Western Blotting

Western blotting assays were conducted according to our previous description [20]. Briefly, cells were collected and lysed in RIPA buffer (Invitrogen, USA), and total proteins were isolated. A BCA assay kit was utilized in order to determine the concentration of the protein, and SDS-PAGE was utilized in order to distinguish the target proteins from the overall protein. Following that, the proteins were deposited onto PVDF membranes (Millipore). Bovine serum albumin (Gibco, USA) was used to block the PVDF membrane for 2 h at room temperature, followed by incubation with primary antibodies against STAT3 (1 : 1000, Abcam) or GAPDH (1 : 2000, Abcam) at 4°C overnight and then with antirabbit HRP secondary antibodies (1 : 1000, Abcam) at 37°C for 1 h. Detection was performed with enhanced chemiluminescence (ECL) reagents. ImageJ software was utilized in order to do statistical analysis and quantify the obtained data. For the purpose of determining relative protein expression, the ratio of densitometry readings to the values that correspond to GAPDH was utilized.

### 2.6. Quantitative Real-Time PCR

RT-PCR was performed as previously described [21]. Using TRIzol reagent (Sigma, USA) and following the technique provided by the manufacturer, total RNA was extracted from a variety of treated cell types. We utilized SuperScript II reverse transcriptase to create cDNA from these samples after we extracted DNA from them (Sigma, USA). Using a LightCycler 480 II PCR machine and SYBR Green PCR master mix, RT-PCR was carried out (Roche). The 2^-*ΔΔ*CT^ approach was utilized in order to standardize the expressions of miR-106a, miR-20b, and STAT3 in relation to those of the internal control U6 or GAPDH. The primer sequences designed by miRprimer are listed in Table 1 [22].

### 2.7. Cytokine Detection by ELISA

The cytokine levels of IL-12, IL-6, TNF-*α*, IL-10, and IFN-*γ* collected from the cell supernatants in each group were analyzed using an ELISA kit (PeproTech, USA) following the manufacturer's instructions.

### 2.8. Target Gene Prediction of miR-106a/20b

Bioinformatics was responsible for carrying out the computational analysis of the forecast. The interactional binding sites between the 3′-UTR of STAT3 mRNA and potential miRNAs were predicted with TargetScan 7.1 (http://www.targetscan.org/), miRDB (http://mirdb.org/), miRMap (https://mirmap.ezlab.org/), miRTarBase (http://mirtarbase.mbc.nctu.edu.tw/), and miRanda (http://www.microrna.org/).

### 2.9. Dual-Luciferase Assay

Oligonucleotides needed for the production of the luciferase reporter vector were designed based on the expected binding site of the 3′-untranslated region of the STAT3 mRNA. A DNA fragment that was located in the middle of the expected binding site was subjected to PCR amplification, and the resultant product was inserted into the pmiR-RB-ReportTM vector between the Not-I and Xho-I restriction sites (RiboBio Co., Ltd., Guangzhou, China). Oligonucleotides of the wild-type version (wt) and mutated version (mut) of the predicted binding site were constructed (RiboBio Co., Ltd., Guangzhou, China). 293 T cells were cultured in DMEM and cotransfected with 50 ng of empty reporter (vehicle), pmiR-RB-STAT3-wt or pmiR-RB-STAT3-mut, and 50 nM negative control miRNA, miR-106a mimic (no. miR10000385; RiboBio Co., Ltd., Guangzhou, China), or miR-20b mimic (no. miR10003187; RiboBio Co., Ltd., Guangzhou, China) using Lipo6000TM (Beyotime, China). After a waiting period of twenty-four hours, the cells were tested with the Dual-Luciferase Assay System to determine whether or not they contained firefly luciferase activity and Renilla reniformis luciferase activity (Promega, USA).

### 2.10. Transient Transfection

Guangzhou RiboBio Co., Ltd., China was responsible for the synthesis and purification of the miR-106a/20b mimics, miR-106a/20b inhibitors, negative control (NC), small interfering RNA (siRNA) for STAT3 (si-STAT3), and negative control siRNA (si-NC). They were transfected into DCs with the use of the lipofectamine 3000 reagent (Invitrogen, USA), following the methods provided by the manufacturer. After 24 hours, total RNA and protein were extracted, and the cells were transfected in Opti-MEM that had been preheated to 37 degrees Celsius and contained 5% carbon dioxide. The medium was replaced after 6 hours. The efficiency of transfection was measured by analyzing miR-27a levels via RT-qPCR.

### 2.11. Cell Counting Kit-8(CCK-8) Measurement

A CCK-8 assay (Toyobo Co., Ltd., Japan) was used to detect the proliferation of T-lymphocytes stimulated by DCs from different transfection groups according to the manufacturer's instructions. GL261 cells were placed into 96-well plates at 1 × 10^5^ cells/ml. After normal cultivation for 24 h, inoculated DC/T fusion cells (1 : 5) with different effect–target ratios (5 : 1, 10 : 1, 20 : 1, 30 : 1) were distributed into Transwell 96-well plates. After 24 h of coculture, the supernatant was discarded. Then, 100 *μ*L of fresh medium (containing 10% CCK-8) was added to each well, and after incubating at 37°C for 2 h in a humidified CO_2_ incubator, the absorbance at 450 nm was measured. Each experiment was repeated 3 times.

### 2.12. EdU Incorporation Assay

In order to conduct the 5-ethynyl-20-deoxyuridine (EdU) incorporation assay, cells were analyzed with a Cell-Light EdU Apollo 488 In vitro Imaging Kit (RiboBio, China) in accordance with the methods provided by the manufacturer. Briefly, in a shaker, the cells were exposed to 50 mM EdU for two hours before being rinsed with glycine at a concentration of 2 mg/mL for five minutes. Then, following permeabilization with 0.3 percent Triton X-100 and staining with Apollo fluorescent dyes, the cells were fixed with 4% paraformaldehyde. In order to stain the cell nuclei, DAPI (Sigma, USA) was incubated with the cells for ten minutes. Using a fluorescent microscope (OLYMPUS IX71), the researchers counted the amount of EdU-positive cells present in each of the five random fields.

### 2.13. In Vivo Tumourigenicity Assay

GSCs (1 × 10^6^ cells/ml) suspended in serum-free DMEM (1 : 1) were subcutaneously injected into the right flanks of C57BL/6 mice. The mice were maintained for six weeks for tumour formation and treatment with DC immunotherapy based on our previous research [4]. After inoculation, tumour sizes were measured, and the body weights were recorded each week after the tumour cell injection. Using bioluminescence imaging, we were able to confirm the presence of tumours (BLI). After a week, the experimental group mice received an intraperitoneal injection of miR106a/20b-mimic-transfected DCs (0.2 mL, total inoculum = 106 cells) every other day for 4 weeks. The control group was injected with the same volume of saline. The mice were anaesthetized and killed 6 weeks after inoculation, and the tumours were removed, weighed, and sectioned.

### 2.14. Statistical Analysis

Each experimental data point was repeated at least three times. The mean values (mean ± SD) are presented. Statistical analysis was performed by GraphPad Prism software 8.0 (San Diego, CA) and SPSS 23.0 (IBM, Corp, Armonk, NY). For the purpose of analyzing the differences between various groups, a one-way analysis of variance (ANOVA) and the least significant difference (LSD) were utilized. Differences were considered statistically significant when *p* was less than 0.05.

## 3. Results

### 3.1. GSC-Enriched Spheroid Acquisition and Antigen Preparation

**Figure 1:**
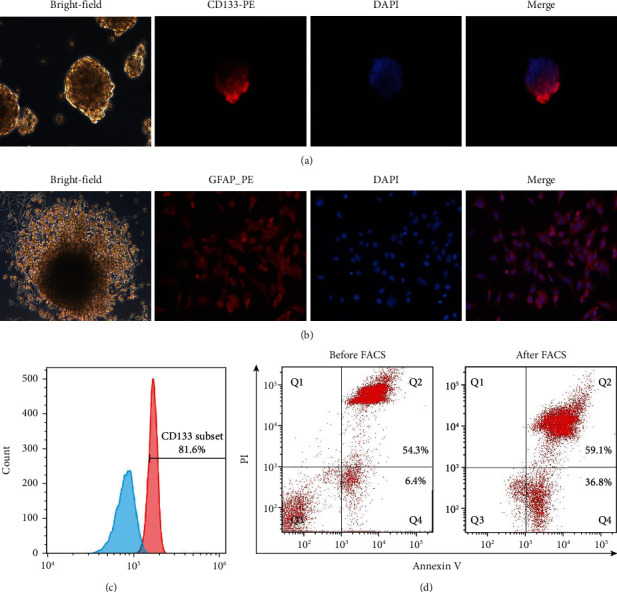
Characterization of GL261-induced cancer stem cells. (a) Tumour spheres expressing CD133 and DAPI. (b) After 3 days of culture in FBS-containing medium, spheres began to spread into adherent monolayers cells. (c) The results of flow cytometry of tumour spheres at the third passage. Red and blue profiles, respectively, represent test group and negative control; number indicates percentage of CD133+ cells. (d) The apoptosis rate was 54.3% + 6.4% before FACS, and 59.1% + 36.8% after FACS (Q3 and Q4).

We cultured GL261 cells in serum-free medium supplemented with EGF, bFGF, and B27 to obtain GSCs from GL261 cells. After 5 days, tumour spheres were visible in the medium. immunocytochemistry assays showed strong fluorescence signals of CD133 in these tumour spheres (Figure 1(a)). When switched to FBS-containing medium, these tumour spheres began to adhere to the bottom of the culture dishes and initiate differentiation, spreading gradually into monolayers and expressing the marker of neuroglial cells, GFAP (Figure 1(b)). Moreover, the accurate proportions of CD133+ cells within GL261 tumour spheres were also detected by flow cytometry. The proportion of CD133+ cells within GL261 tumour spheres reached 81.6% at P3 (Figure 1(c)). Then, we extracted GSC antigens after processing with apoptosis inducers, labelled them with Annexin V-FITC/PI and subjected them to fluorescence-activated cell sorting (FACS), the apoptosis rate of the GSC antigens reached 59.1% + 36.8% (Q3 and Q4) as determined by FACS (Figure 1(d)).

### 3.2. BMDCs Loaded with GSC Antigens by the Apoptotic Method

**Figure 2:**
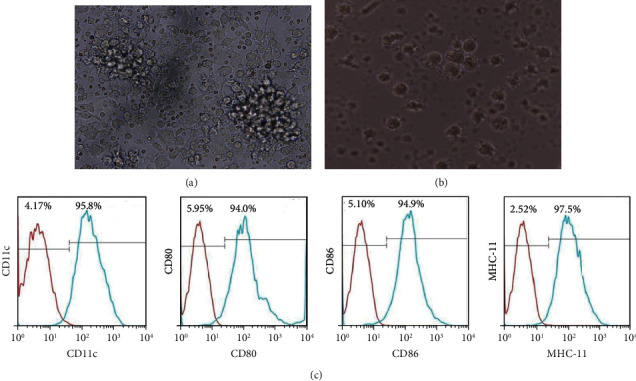
Dendritic cell isolation and characterization. (a and b) The morphology of the immature DCs and mature DCs (200x magnification). (c) The expression of DC markers before and after maturation. The green lines represent the expression of LPS-induced DCs receptors; the red lines represent negative controls.

DCs were isolated from C57BL/6 mouse bone marrow. These imDCs were able to form colonies after 3 days of culture (Figure 2(a)). After LPS stimulation, these imDCs started to mature and developed abundant dendritic structures (Figure 2(b)). The quality of the DCs was characterized by cell surface marker analysis, and the results are displayed in Figure 2(c) revealed that LPS-induced DCs expressed significantly higher levels of DC mature markers, including CD11c, CD80, CD86, and MHC-II, than imDCs. On day 5, bone marrow-derived imDCs were treated for 24 h with apoptotic GSC antigens at a 3 : 1 (stimulator : responder) ratio. Meanwhile, untreated DCs were used as a negative control.

### 3.3. Expression and Activation of STAT3 Is Elevated in DCs

**Figure 3:**
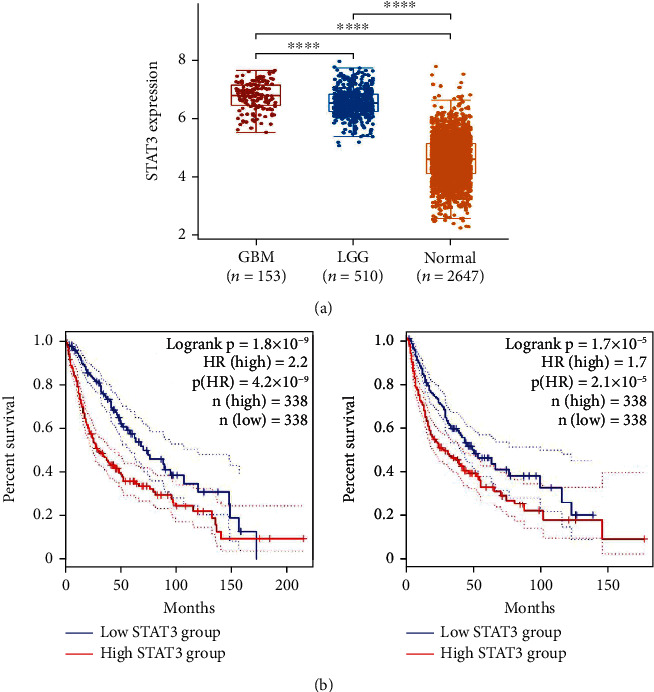
The expression of STAT3 and its prognostic value in glioma. (a) The expression of STAT3 was distinctly increased in GBM and LGG specimens compared with nontumour specimens based on TCGA datasets. (b) Patients with high STAT3 expression showed a shorter OS and PFS than those with low STAT3 expression in patients with GBM and LGG based on TCGA datasets.∗∗∗∗*p* < 0.0001.

In this study, we analyzed STAT3 expression in GBM (glioblastoma multiforme) and LGG (low-grade glioma) tissues from the TCGA microarray database and found that STAT3 expression significantly increased, which was associated with lower overall survival and disease-free survival (Figures 3(a)–3(b)).

### 3.4. Inhibition of STAT3 Expression by a Specific siRNA Promoted the Maturation of DCs Loaded with GSC Antigens

**Figure 4:**
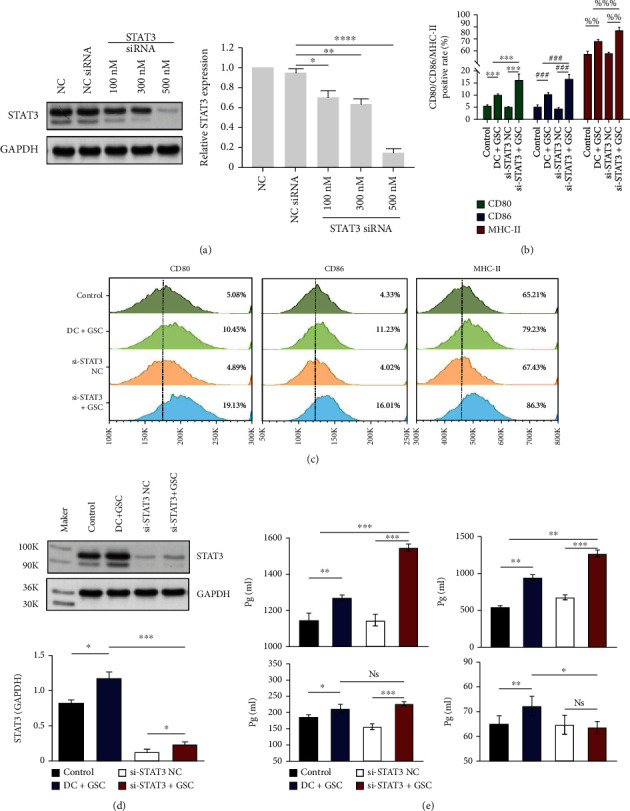
The expressions of DC cell surface molecules (CD80, CD86, and MHC-II) as determined by flow cytometric analysis. (a) DCs were transfected with 500 nM nonspecific siRNA (NC-siRNA) or 100 nM, 300 nM, or 500 nM siRNA targeted against STAT3 (STAT3 siRNA). (b and c) Flow cytometry analysis of changes in the expression of costimulatory molecules and mature molecules on the cell surface of different DC groups. (d) The expression of STAT3 in each DC groups by Western blotting. (e) The cytokine secretion levels of IL-12, IL-6, TNF-*α*, and IL-10 in the supernatants were measured in the four groups by ELISA. The levels of IL-12 and IL-6 were significantly increased in the si-STAT3+GSC group compared with the DC+GSC group, and the level of IL-10 showed the opposite trend. Molecular weight: STAT3(88 kDa) and GAPDH(36kD). ^∗^*p* < 0.05, ^∗∗^*p* < 0.01, ^∗∗∗^*p* < 0.001.

Given the association of STAT3 expression with DC immune infiltration levels in LGG and GBM, we next evaluated the stimulating effects of STAT3 on imDCs loaded with GSC antigen via secretion and expression of surface markers of DCs using ELISA and flow cytometry assays. A small interfering RNA (siRNA) was designed to induce gene silencing of STAT3 in DCs (Si-STAT3 DCs). In a manner that was dependent on concentration, the transfection of STAT3 siRNA distinctly suppressed the expression of STAT3. The effects of STAT3 siRNA were specific in the sense that it was unable to suppress the production of GAPDH, a protein that is unrelated to STAT3 (Figure 4(a)).

In order to investigate the maturation process of DC, flow cytometry was utilized for the purpose of detecting the expression of costimulatory markers CD80, CD86, and MHC-II. The results showed that BMDCs induced in vitro using GM-CSF were typically immature. Upon recognition of apoptotic GSC antigens, DCs upregulated the expressions of CD80, CD86, and MHC-II. Interestingly, the STAT3-deficient DCs, transfected with Si-STAT3 for 24 h were more sensitized to GSCs, as they expressed higher expressions of CD80, CD86, and MHC-II than DC+GSCs (19.13 ± 2.41% vs. 10.45 ± 0.56%, 16.01 ± 0.41% vs. 11.23 ± 0.79%, and 86.30 ± 2.38% vs. 79.23 ± 1.53%, respectively). The statistically significant differences in expression are shown (Figures 4(b)–4(c)).

In vitro GSC stimulation of imDCs and STAT3-null DCs induced the secretion of IL-12, IL-6, TNF-*α,* and IL-10 into the supernatant, which was quantified by ELISA. We found that imDCs exhibited little cytokine secretion. DCs loaded with apoptotic GSC antigens secreted higher levels of IL-12, IL-6, TNF-*α,* and IL-10 than imDCs. Statistically significant differences are displayed in the corresponding figures (Figure 4(d)). After transfection with Si-STAT3 for 24 h, the STAT3-deficient DC group produced significantly more amount of proinflammatory IL-12 and IL-6, but less IL-10 than the DC+GSCs, the secretion levels of TNF-*α* were not substantially increased (Figure 4(e)).

### 3.5. Analysis of the Role of STAT3 Signalling in T Cell Differentiation and IFN-*γ* Release

**Figure 5:**
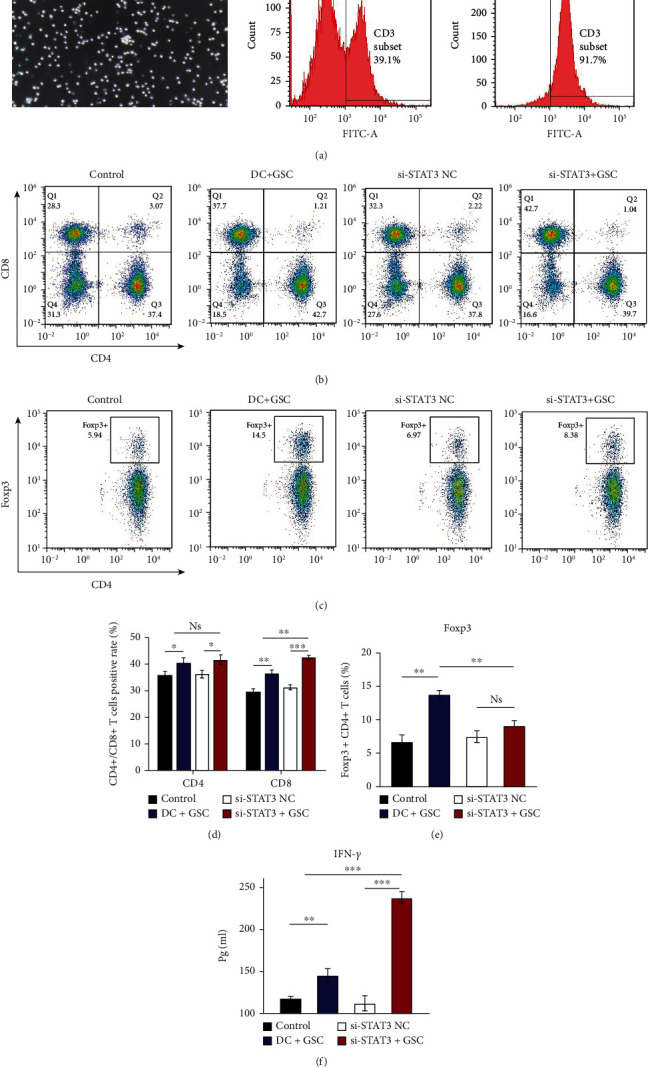
Isolation, differentiation, and cytokine secretion of T-lymphocytes. (a) The image of T cells (100x) and the expression of T cell marker CD3-FITC before and after magnetic-activated cell sorting (MACS). Numbers indicate percentages of positive cells. (b and d) CD8+ T-cell generation in the STAT3 deficiency DC group was significantly increased. (c and e). Foxp3+ CD4+ T-cell generation in the STAT3 deficiency DC group had evaluated less than other groups. (f) ELISA results showed that secretion of INF-*γ* by T-lymphocytes stimulated with STAT3-null DCs pulsed with GSCs was upregulated compared with that by T-lymphocytes stimulated with DCs transfected with GSCs (1.63-fold). Bars represent the mean ± SD. Comparison with the T-lymphocytes group stimulated with DCs loaded GSCs. ^∗^*p* < 0.05, ^∗∗^*p* < 0.01, ^∗∗∗^*p* < 0.001.

Further experiments were conducted to examine the differentiation of T cells induced by DCs after pulsed with GSC antigens using flow cytometry. First, we isolated T cells from C57/BL6 mouse splenocytes. According to the results of MACS analysis of T cells, more than 90% of the T cells could be purified from the splenocytes of C57BL/6 mice (Figure 5(a)). Then, T cells were plated into 96-well plates at a density of 1 × 10^4^ cells/well to be cocultured with each DC group at a 1 : 5 ratio. Flow cytometry analysis showed that the proportion of CD8-positive T cells was significantly increased in the STAT3-deficient DC group (si-STAT3+GSC) compared to the DC+GSC group (42.70 ± 0.54% vs. 37.70 ± 1.02%, *p* < 0.05). However, the proportion of CD4-positive T cells was not significantly different between the two groups (39.70 ± 1.57% vs. 42.70 ± 1.51%, *p* > 0.05) (Figures 5(b)–5(d)). Interestingly, the number of Foxp3-positive CD4-positive Treg T cells generated in the si-STAT3+GSC group was markedly lower than that in the DC+GSC group (8.38 ± 0.62% vs. 14.50 ± 0.50%, *p* < 0.05) (Figures 5(c)–5(e)).

Furthermore, the DC/T cell coculture supernatants were collected for cytokine analysis by ELISA. The IFN-*γ* release results showed that the cytokine secretion capacity of T cells treated with STAT3-deficient DCs was significantly stronger than that of the DC+GSC group (238.24 ± 5.78% vs. 145.53 ± 6.35%, *p* < 0.05) (Figure 5(f)). The results demonstrated that the capacities of STAT3-deficient fusion-DCs to stimulate cytotoxic lymphocyte T cell (CTL) differentiation were stronger than those of the other groups and reduced the immune tolerance of helper T cells.

### 3.6. Prediction and Identification of miR-106a/20b Targeting of the STAT3 Gene

**Figure 6:**
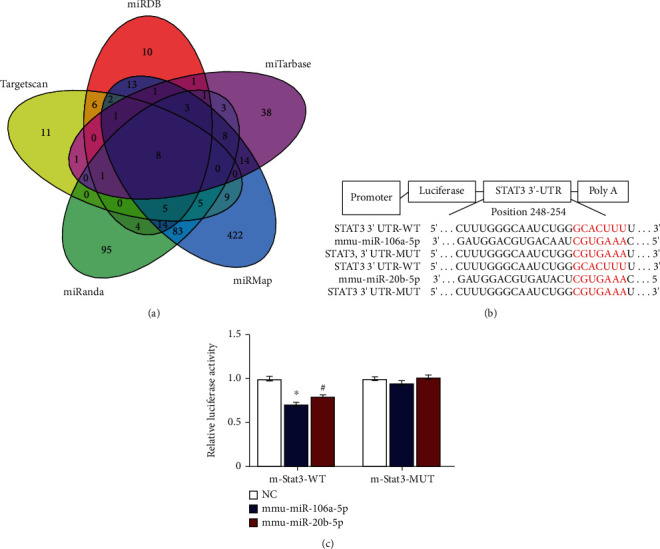
Identification of miR-106a/20b, which regulates STAT3 expressions. (a) The predicted consequential pairing between the miRNA (miR-106a and miR-20b) and STAT3 3′-UTR; (b and c) Relative luciferase activity upon cotransfection of the empty reporter (vehicle) with pmiR-RB-STAT3-WT or pmiR-RB-STAT3-MUT and the control miRNA, miR-106a, or miR-20b mimic. ^∗∗∗^*p* < 0.001.

To further explore the molecular regulatory mechanism of GSC on DCs, we identified the upstream regulatory miRNA of STAT3 by using TargetScan, miRDB, miRTarBase, miRMap, and miRanda, and predicted eight miRNAs that may target the STAT3 3′-UTR (Figure 6(a)), and six of which belong to the miR-17 family, namely, the miR-17-92 cluster (miR-17-5p, miR-20a-5p), miR-106b-25 cluster (miR-106b-5p, miR-93-5p), and miR-106a-363 cluster (miR-106a-5p, miR-20b-5p)(Table 2). The miR-106a-363 cluster is a highly conserved miRNA cluster on the human X chromosome in humans that is abundant 7in plasma and serum. TargetScan showed that miR-106a and miR-20b were the same and were conserved from nucleotide positions 248 to 254, which had only one target site on the STAT3 mRNA 3′-UTR (Figure 6(b)). Both of their context+ score percentiles were 95, and the PhastCons score was also the same–0.6361. The mirSVR score of the two miRNAs showed a minor difference that of miR-106a was -0.8717, and that of miR-20b was -0.898 (Table 2). This demonstrated that the two miRNAs were very complimentary to the locations that they were aiming for.

To determine whether or not miR-106a/20b can control the expression of STAT3, a dual-luciferase test was carried out. The relative luciferase activity from pmiR-RB-STAT3-WT was inhibited by the miR-106a (0.76 ± 0.31%) or miR-20b (0.80 ± 0.36) mimic. Instead, the miR-106a (0.95 ± 0.63) or miR-20b (1.02 ± 0.41, *p* > 0.05) mimic did not exhibited a distinct role (Figure 6(c)).

### 3.7. Analysis of the Relationship between miR-106a/20b and STAT3 Expression in DCs

**Figure 7:**
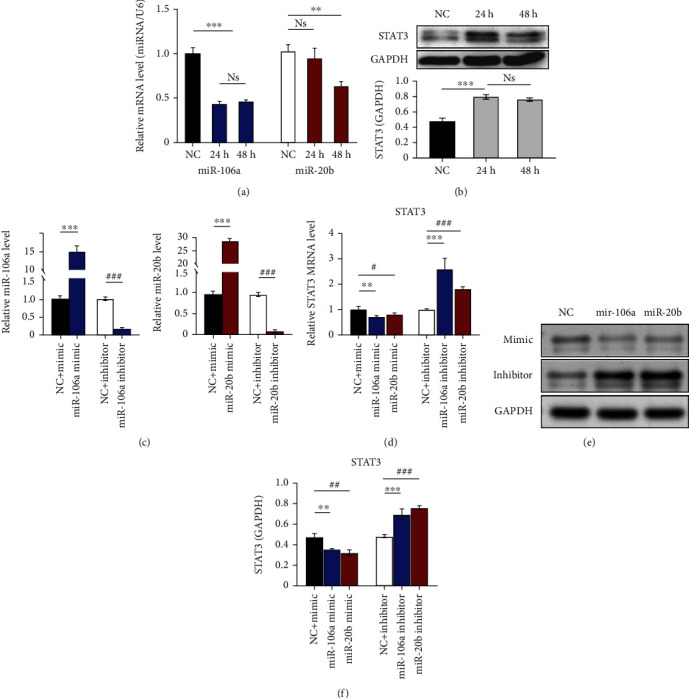
Relative expression levels of miR-106a, miR-20b, and STAT3 mRNA in DCs after loading with GSCs. (a) miR-106a/20b expression was determined in the NC group and after 24 and 48 h of GSC stimulation by real-time PCR. (b) STAT3 mRNA expression was determined in the NC group and after 24 and 48 h of GSC stimulation by Western blotting. (c) Relative expression levels after miR-106a/20b mimic or inhibitor transfection in DCs. (d) RT-PCR of STAT3 expressions in each DC-transfected group after 12 h of GSC stimulation. (e and f) Western blotting analysis of STAT3 protein expression in each DC-transfected group. ^∗∗^*p* < 0.01, ^∗∗∗^*p* < 0.001.

RT-qPCR was applied to examine miR-106a and miR-20b, and Western blot was applied to test STAT3 mRNA expressions during the maturation of DCs pulsed with GSCs. If miR-106a and miR-20b expression in the normal control DC group (NC) was defined as 1, the expression level of miR-106a after 24 h in DC-loaded GSCs was 0.44 ± 0.02 lower and maintained at 0.47 ± 0.02 after 48 h. Although the expression of miR-20b after 24 h was not significantly changed, it also decreased significantly to 0.64 ± 0.05 after 48 h (Figure 7(a)). Correspondingly, the STAT3 mRNA expression level in DCs stimulated with GSCs was 1.67 ± 0.79-fold higher than that in the control group after 24 h and remained 1.60 ± 0.15-fold higher after 48 h (Figure 7(b)).

The results of RT-PCR revealed that the expressions of miR-106a/20b (15.66 ± 0.76/29.80 ± 2.10) were higher in the miR-106a/20b mimic groups. The expressions of miR-106a/20b (0.17 ± 0.03/0.08 ± 0.04) was lower in the miR-106a/20b inhibitor groups than in the inhibitor control group (NC-inhibitor) (Figure 7(c)). After 24 h of GSC stimulation, STAT3 expression was determined using real-time PCR and Western blotting. The STAT3 mRNA expressions in both the miR-106a-mimic transfected group (0.71 ± 0.06, *p* < 0.05) and the miR-20b-mimic transfected group (0.81 ± 0.13, *p* < 0.05) were significantly lower. In contrast, the levels in both the miR-106a-inhibitor transfected group (2.60 ± 0.44, *p* < 0.05) and the miR-20b-inhibitor transfected group (1.40 ± 0.08, *p* < 0.05) were significantly higher than those in the NC-inhibitor group (Figure 7(d)). Alternatively, the expression of the STAT3 protein in the miR-106a-mimic group (0.36 ± 0.01, *p* < 0.05) and the miR-20b-mimic group (0.32 ± 0.02, *p* < 0.05) was distinctly decreased compared with that in the NC-mimic group (0.58 ± 0.02), as shown by Western blotting. However, the expression trend of the STAT3 protein in the miR-106a/20b-inhibitor groups was the opposite (Figures 7(e) and 7(f)). These results show that the expression of STAT3 in DCs loaded with GSC antigen was negatively related to the levels of miR-106a/20b.

### 3.8. Mir-106a/20b Enhanced the Maturation of DCs and the Activity of T Cells In Vitro

**Figure 8:**
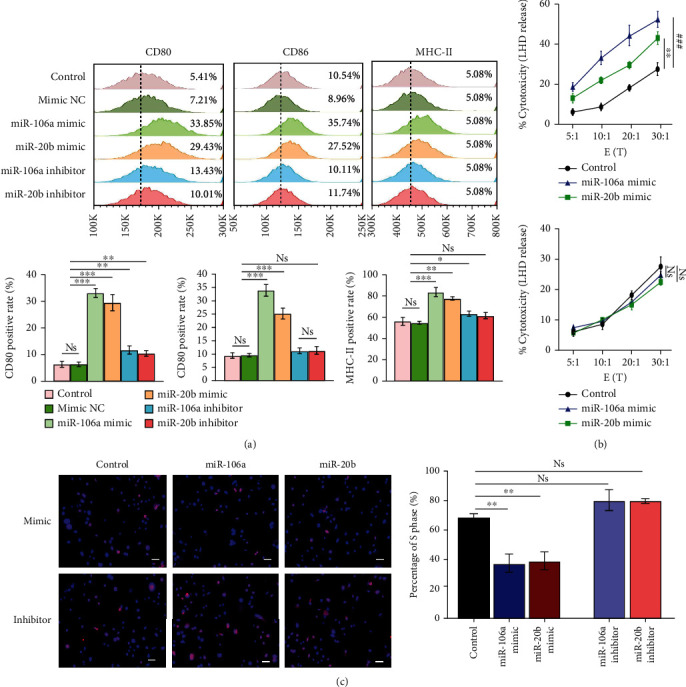
The effect of miR-106a/20b levels on the maturation of DCs and the cytotoxicity of induced T-lymphocytes against GSCs in each group. (a) The expressions of CD80, CD86, and MHC-II in the miR-106a/20b mimic group was distinctly higher than that in the control DC group by Flow cytometry analysis. (b) LDH release assay was applied for the detection of the cytotoxicity of T cells in the miR-106a/20b mimic group; substantially higher cytotoxicity against GSCs relative to the control group at different E/T ratios was observed. (c) GSC proliferation was determined by EdU staining. ^∗∗^*p* < 0.01.

Next, we evaluated the expression of surface markers (CD80, CD86, and MHC-II) in DCs transfected with miR-106a/20b mimics and inhibitors using flow cytometry assay after incubation with GSCs at a ratio of 3 : 1 for 48 h. Our findings revealed that the expressions of CD80, CD86 and MHC-II were distinctly higher in the miR-106a/20b mimic-transfected DC groups than in the normal DC group. (∗*p* < 0.05, Figure 8(a)). In vitro, we used lactate dehydrogenase (LDH) release to assess the cytotoxicity of T cells against the tumour cells induced from the third passage of GSCs at E:T (effector: target cell) ratios of 5 : 1, 10 : 1, 20 : 1, and 30 : 1. The T cells were cocultured with DCs transfected with miR-106a/20b mimics and inhibitor in the upper chamber of a 24-well Transwell plate, and the tumour cells were cultured in the lower chamber of the Transwell plate at 10^4^ cells/ml until 80% confluency was reached. As shown in Figure 8(b), the cytotoxicities against GSCs at E/T ratios of 10 : 1, 20 : 1, and 30 : 1 in the miR-106a/20b mimic-transfected DC/T cell coculture groups were higher than those in the mimic control group (∗*p* < 0.05), but the T cells showed a trend towards lower cytotoxicity at each of the different E/T ratios in the miR-106a/20b inhibitor-transfected groups compared with the inhibitor control groups, although this difference was not statistically significant (∗*p* < 0.05)).

The effects of T cells cocultured with DCs transfected with miR-106a/20b mimics on GSC proliferation were examined via an EdU assay. We found that the inhibition ability to GSCs of the stimulated T cells in the miR-106a/20b mimic-transfected DC groups was significantly higher than that in the control DC/T fusion group (*p* ≤ 0.001) and the miR-106a/20b inhibitor-transfected DC/T groups (*p* ≤ 0.001) at a 20 : 1 E/T ratio ∗*p* < 0.05, Figure 8(c)). In summary, miR-106a/20b mimic transfection increases the maturation of DCs, and significantly enhances the tumour cytotoxicity of cocultured T cells in vitro compared with those incubated with normal DCs.

### 3.9. Mir-106a/20b Enhanced the Tumour Suppression Ability of DCs In Vivo

**Figure 9:**
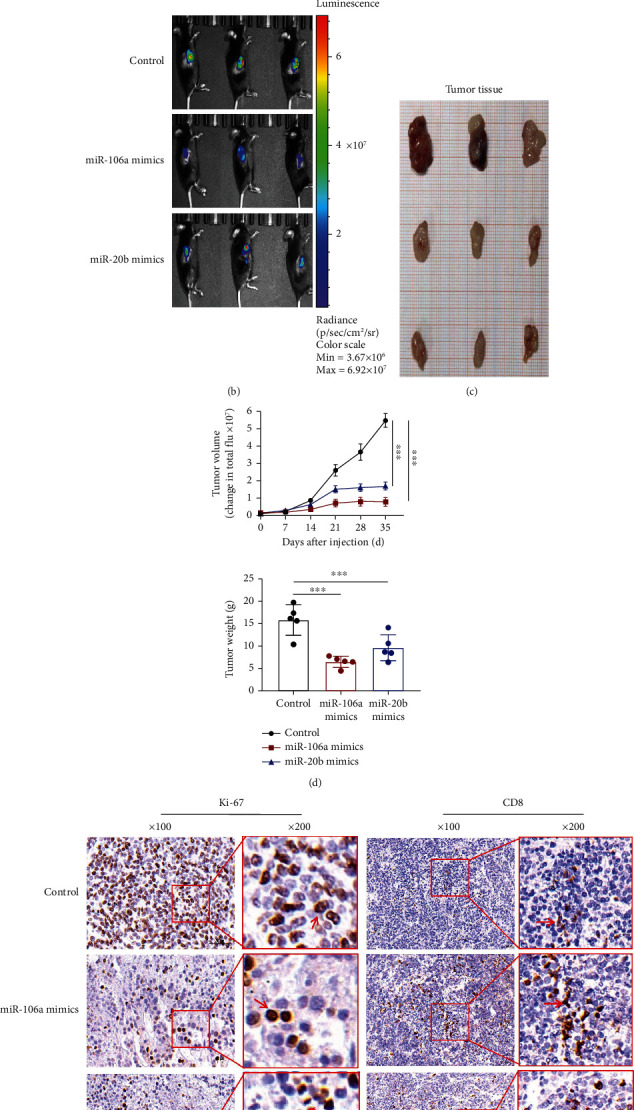
miR-106a/20b mimics increased the antitumour characteristics of DCs and inhibited tumour growth in an immunocompetent syngeneic GSC model. (a) Treatment scheme for GSC-implanted mice. (b) Representative BLI images showing the final tumour volume; (c and d) Measurements of tumour volume and weight. (e) Immunohistochemistry of tumour sections stained with Ki-67 antibodies shows cellular proliferation of the tumour cells.(100x; the arrows indicate the Ki67-positive cells.). CD8+ T cell infiltration into the tumour as determined by IHC (arrows indicate the CD8-positive cells). (The red boxes indicate the details at higher magnification (200x).) ^∗∗∗^*p* < 0.001.

Finally, the study evaluated the effect of DCs transfected with miR-106a/20b mimics on the GSC-induced tumours in C57BL/6 mice. For the subcutaneous tumour of the mouse model, Luc-tagged GSCs (1 × 10^6^) were injected subcutaneously into the right back near the hind leg of C57BL/6 mice. An overview of the treatment scheme for the GSC model is shown in Figure 9(a). It was observed that the miR-106a/20b mimic-transfected DC treatment caused the formation of tumours with smaller sizes and lower weights (Figuress 9(b)–9(d)) compared with the control group (*p* < 0.01). Immunohistochemical measurement of the expression of Ki-67 and CD8+ T-cell-specific antibodies revealed suppressed cellular proliferation and an increased infiltration of tumours in mice treated with the miR-106a/20b mimic compared to the control (Figure 9(e)). Our data revealed that miR-106a/20b had a tumour-suppressing effect and increased the effectiveness of immunotherapy.

## 4. Discussion

Glioma is the most common malignant primary central nervous system tumour in adults and generally has poor prognosis. In past studies, CSCs, including GSCs, have been demonstrated in several tumours, such as pancreatic cancer, colon cancer and melanoma [23]. Due to these stem cell-like characteristics, GSCs in glioma are thought to be responsible for tumour initiation, progression, and resistance to chemoradiotherapy, contributing to relapses after treatment [24]. In recent years, DC-based immunotherapy has emerged as a promising strategy to treat various cancers. DCs are APCs and trigger the signalling pathway that leads to adaptive and innate immunity via modulating the activation and differentiation of T cells through a series of receptor recognition-related pathogenic products [25]. However, the antitumour immunotherapy effect of DC vaccine-based targeting of glioma cells remains unsatisfactory due to the poor immunogenicity of tumour cell antigens [26]. Therefore, targeting GSCs and developing therapies that target the GSC population represent attractive therapeutic strategies of DC-based immunotherapy for glioma. Based on this, the main aim of this study was to activate immunotherapy using DC vaccines loaded with GSC-derived antigens to initiate T-cell-mediated antitumour immunity.

Cancer immunotherapy requires both tumour-specific antigen presentation in DCs and sufficient immune stimulatory responses. The stage of development of DC plays a crucial role in the process of developing immunological responses in response to vaccination based on DC [27], where it is of the utmost importance to achieve an increase in the expression of critical costimulatory and maturation molecules (such as CD80, CD86, and MHC-II) that are involved in the activation of effector T cells [28]. Previous studies have shown that DC-based vaccination strategies have often used total antigen lysates derived from GSCs, such as heat-treated, freeze-thawed, and dying cells (apoptotic bodies and necrotic cells) [29–31] or cells fused directly with tumour cells by electroporation [32], which have been shown to be safe and effective. In this study, we chose apoptotic GSC-derived antigens to pulse DCs. Under these conditions, the expressions of MHC-II, CD86, and CD80 on the DC surface and the secretion of cytokines, such as IL-12, IL-6, and TNF-*α*, were increased. In addition, it triggered an increase in CD8-positive cytotoxic T cells. Nonetheless, the mechanisms underlying tumour cell escape from immunity remain unclear. Therefore, we attempted to delve into the molecular mechanisms by which the antigen presentation ability of DCs is enhanced to reduce CSC escape from immune surveillance.

Evidence has been presented that the downregulation of inflammasome components by tumour cells favours immune escape by limiting the recruitment and activation of antitumour immune cells [33]. The STAT3-mediated signalling cascade is one of these, and it is a necessary mediator of tumour-induced immunosuppression for immune cell activation. At the same time, it facilitates the transcription of genes that are naturally anti-inflammatory and exhibit immunosuppressive activities in DC maturation [34]. As a consequence of this, STAT3 has come to be regarded as a potentially fruitful target in DCs, and the induction of anticancer immunity that it facilitates may be made more effective by the application of targeted cancer immunotherapy. In our research, our group delve into the roles that STAT3 plays in the differentiation and function of DCs. We first analyzed STAT3 expression in the TCGA and GEO databases, as well as in a glioma tissue microarray, and found that higher expression of STAT3 in tumour tissue predicted a poor prognosis. We also evaluated the correlations of STAT3 expression with immune infiltration levels in GBM and LGG from TIMER, and we discovered that STAT3 expression was positively related to the infiltration levels of macrophages, neutrophils, B cells, CD8+ T cells, and DCs. After discovering that the levels of immune infiltration in GBM and LGG had a positive correlation with the levels of STAT3 expression, we arrived to the conclusion that this is the case. Besides, we determined whether STAT3 deletion may enhance the activity of GSC lysate-pulsed DCs to induce T cell differentiation and immune responses. We observed that STAT3-null DCs expressed high levels of CD80, CD86, and MHC-II and secreted more IL-12 and IL-6 and less IL-10 in vitro. Our data also confirmed that silencing of STAT3 in DCs by siRNA, an alternative to gene inhibition, could attenuated the differentiation of regulatory T cells, increased the number of CD8-positive T cells, and promoted the secretion of IFN-*γ* to activate the cytotoxicity of T cells, which is consistent with previously published reports [35, 36]. All of these results suggested that STAT3 could orchestrate a diverse set of immunosuppressive processes to impact the differentiation of BMDCs. Correcting such defects is favourable for enhancing tumour antigen presentation with DCs.

Nevertheless, there has been limited discussion about how GSC antigens affect STAT3 expression in these imDCs and the immunological tolerance consequences of such perturbations in DC-induced T cells against glioma. Growing researches have shown that miRNAs can act as key tumour regulators, either as cancer suppressors or oncogenes [37]. To study these targets of DCs, we used a miRNA approach for which we screened the levels of miRNAs associated with STAT3. Bioinformatics analysis was conducted by using TargetScan, miRDB, miRMap, miRTarBase, and miRanda. We identified eight miRNAs with target sites in the STAT3 mRNA 3'-UTR. In fact, six of them with high similarity belonged to the miR-17 family, namely, the miR-17-92 cluster (miR-17-5p, miR-20a-5p), miR-106b-25 cluster (miR-106b-5p, miR-93-5p), and miR-106a-363 cluster (miR-106a-5p, miR-20b-5p). The miR-17 family is, thus far one of the best-studied miRNA families, accounting for more than 2.7% of the total dysregulated pool of all miRNAs [38]. Specifically, miR-106a and miR-20b, which are part of the miR-106-363 cluster located on chromosome X in humans, were reported to play an important regulatory role in different types of human cancer, including glioma [39–41]. For example, Pan et al. discovered that by controlling the expression of PAK5, miR-106a was able to prevent renal cell carcinoma cells from migrating and invading other tissues [42]. Hong et al. also found that miR-20b functioned as a tumour suppressor in THCA by regulating the MAPK/ERK signalling pathway [43]. However, in vitro and in vivo studies have shown that miR-106a is an oncogene that promotes human gastric cancer cell growth and metastasis [44]. Zhou et al. found that miR-20b promoted the proliferation of breast cancer cells via modulating PTEN [45].

In the present study, we focused on identifying the roles of miR-106a/20b in DC immunomediation, as there have been few studies on this issue. Our group observed that the expressions of miR-106a and miR-20b in DCs pulsed with GSCs was downregulated and that the STAT3 level was elevated. Transfection with miR-106a-5p and miR-20b-5p mimics downregulated the expression of STAT3 in DCs. These results and those from TargetScan predicted that the two miRNAs were complementary 3'-UTR sequences of target STAT3 mRNA, which was further substantiated the targeting relationship by a dual luciferase reporter gene assay. Furthermore, downregulation of STAT3 in DCs by transfection with miR-106a and miR-20b mimics, increased the expression levels of CD80, CD86, and MHC-II molecules on the DC surface, which are associated with the maturation of DCs. The proliferative viability in vitro and the tumour formation capacity of the mouse tumour cells derived from GSCs in vivo were reduced significantly when the expression of miR-106a/20b increased. Our findings suggested that GSCs can attenuated the function of DCs via the miR-106a/20b-mediated the STAT3 pathway. Upregulation of the miR-106a/20b exerted a positive effect on the maturation and the antigen-presenting ability of DCs and diminished GSC immune escape by silencing STAT3.

There are still several limitations to this study. First, due to the limited number of participants in this study's sample, the data that were analyzed were insufficient to provide a complete picture. It is necessary to collect more prospective clinical data in order to verify the validity of the current data. Secondly, more experiments in vivo and vitro were needed for verification of the results we identified by bioinformatic analysis.

## 5. Conclusion

Overall, despite some limitations of our study, we demonstrated an inverse correlation between the expressions of miR-106a/20b and that of STAT3. GSCs inhibited the maturation of DCs and the killing effects of T cells on glioma cells by decreasing miR-106a/20b levels and consequently regulating STAT3 expression. Therefore, downregulating the expression of STAT3 indirectly via miR-106/20b could serve as a potential new therapeutic method to promote DC maturation and attenuate tumour stem cell proliferation.

## Figures and Tables

**Table 1:** Primers used for quantitative real-time PCR.

Genes	Primer sequence
STAT3	
Forward	5′-GGGCTCTTGTCAGCAATG-3′
Reverse	5′-ACGGTCC AGGCAGATGTT-3′
GAPDH	
Forward	5′-CTGGAGAAACCTGCCAAGTA-3′
Reverse	5′-TC ATACCAGGAAATGAGCTTGAC-3′
Mmu-miR-106a	
Forward	5′-AAAGTGCTTACAGTGCAGGTAG-3′
Mmu-miR-20b	
Forward	5′-CAAAGTGCTCATAGTGCAGGTAG-3′
U6	
Forward	5′-CTCGCTTCGGCAGCACA-3′
Reverse	5′-AACGCTTCACGAATTTGCGT-3′

**Table 2:** Distribution and association of the miRNA with STAT3.

miRNA	miRDB score	Context + score percentiles	mirSVR score	Location
Hsa-miR-17-5p	98.305	96	-0.8717	13q31.32
Hsa-miR-93-5p	98.2034	96	-0.8946	
Hsa-miR-20a-5p	98.0351	96	-0.898	7q22
Hsa-miR-106b-5p	97.9383	96	-0.898	
Hsa-miR-106a-5p	98.2236	95	-0.8717	Xq26
Hsa-miR-20b-5p	98.1139	95	-0.898	

## Data Availability

The datasets used and/or analyzed during the current study are available from the corresponding author upon reasonable request.

## Abbreviations

DCs: Dendritic cells
CNS: Central nervous system
HGG: High-grade glioma
GSCs: Glioma stem cells
APCs: Antigen-presenting cells
CCK-8: Cell counting kit-8
DMEM: Dulbecco's modified Eagle's medium
ECL: Enhanced chemiluminescence
FBS: Foetal bovine serum
HRP: Horseradish peroxidase
M-CSF: Macrophage-colony stimulating factor
PVDF: Polyvinylidene difluoride
SDS–PAGE: Sodium dodecyl sulfate-polyacrylamide gel electrophoresis
IFN-*γ*: Interferon-*γ*
FACS: Fluorescence-activated cell sorting
ELISA: Enzyme-linked immunosorbent assays
siRNA: Small interfering RNA
UTR: Untranslated region.
